# Restoration Enhances Wetland Biodiversity and Ecosystem Service Supply, but Results Are Context-Dependent: A Meta-Analysis

**DOI:** 10.1371/journal.pone.0093507

**Published:** 2014-04-17

**Authors:** Paula Meli, José María Rey Benayas, Patricia Balvanera, Miguel Martínez Ramos

**Affiliations:** 1 Natura y Ecosistemas Mexicanos A.C., México DF, México; 2 Departamento de Ciencias de la Vida, Universidad de Alcalá, Alcalá de Henares, Madrid, Spain; 3 Centro de Investigaciones en Ecosistemas, Universidad Nacional Autónoma de México, Morelia, Michoacán, México; University of Guelph, Canada

## Abstract

Wetlands are valuable ecosystems because they harbor a huge biodiversity and provide key services to societies. When natural or human factors degrade wetlands, ecological restoration is often carried out to recover biodiversity and ecosystem services (ES). Although such restorations are routinely performed, we lack systematic, evidence-based assessments of their effectiveness on the recovery of biodiversity and ES. Here we performed a meta-analysis of 70 experimental studies in order to assess the effectiveness of ecological restoration and identify what factors affect it. We compared selected ecosystem performance variables between degraded and restored wetlands and between restored and natural wetlands using response ratios and random-effects categorical modeling. We assessed how context factors such as ecosystem type, main agent of degradation, restoration action, experimental design, and restoration age influenced post-restoration biodiversity and ES. Biodiversity showed excellent recovery, though the precise recovery depended strongly on the type of organisms involved. Restored wetlands showed 36% higher levels of provisioning, regulating and supporting ES than did degraded wetlands. In fact, wetlands showed levels of provisioning and cultural ES similar to those of natural wetlands; however, their levels of supporting and regulating ES were, respectively, 16% and 22% lower than in natural wetlands. Recovery of biodiversity and of ES were positively correlated, indicating a win-win restoration outcome. The extent to which restoration increased biodiversity and ES in degraded wetlands depended primarily on the main agent of degradation, restoration actions, experimental design, and ecosystem type. In contrast, the choice of specific restoration actions alone explained most differences between restored and natural wetlands. These results highlight the importance of comprehensive, multi-factorial assessment to determine the ecological status of degraded, restored and natural wetlands and thereby evaluate the effectiveness of ecological restorations. Future research on wetland restoration should also seek to identify which restoration actions work best for specific habitats.

## Introduction

Wetlands harbor significant biodiversity [1] and supply crucial ecosystem services (ES) [2], [3], which are defined as the benefits that people obtain from ecosystems [4]. ES provided by wetlands include regulating water purification, protecting the ecosystem from soil erosion and effects of flooding, and nursing the early growth of many species essential to oceanic fisheries (**Table 1**). Although wetlands occupy less than 9% of the Earth's terrestrial surface, they contribute up to 40% of global annual renewable ES [5]. Despite their importance to human societies, wetlands are rapidly being degraded and destroyed [5], threatening the ecosystem and biodiversity on which wetland ES depend.

**Table 1:** Principal ecosystem services (ES) supplied by wetlands.

*ES type* 1	*Individual ES*	*Description*
	Biogeochemical cycling	Maintenance of natural exchange or flux of material and energy between living and nonliving components of biosphere, thereby supporting climatic and biological dynamics.
Supporting	Biotic interactions	Pollination of wild species or crops; seed dispersal; preservation and maintenance of trophic chains.
	Habitat (terrestrial)	Habitat for resident and transient terrestrial populations (refugia/nursery).
	Habitat (aquatic)	Habitat for resident and transient aquatic populations (refugia/nursery).
	Plant food/raw material	The proportion of gross primary production that can be extracted as food or raw materials.
Provisioning	Animal food/raw material	The proportion of secondary production that can be extracted as food or raw materials.
	Water supply	Filtering, retention and storage of fresh water for human use (domestic, industrial, agriculture).
	Climate regulation	Regulation of the chemical composition of the atmosphere, global temperature, and other biologically mediated climatic processes at global and regional levels.
	Hydrological dynamics	Regulation of natural hydrological flows, role of land cover in regulating runoff and river discharge, and infiltration; groundwater recharge.
Regulating	Water quality	Retention and removal or breakdown of xenic nutrients and compounds; water purification.
	Regulation of extreme events	Capacity and integrity of ecosystem response to environmental fluctuation such as floods or storms, or to other extreme events.
	Regulation of soil fertility and erosion	Soil maintenance and formation, for both natural ecosystems and crops; sediment retention and prevention of erosion; shoreline stabilization; accumulation of organic matter.
	Regulation of invasive species, pests, and diseases	Regulation of invasive species populations; trophic-dynamic regulations of pest populations.
	Cultural	Contribution by ecosystems to experiences that benefit human population directly or indirectly.
Cultural	Recreation	Provision of opportunities for recreational activities.

1 MEA (2005).

To compensate for their extensive degradation, wetland restoration has become common practice around the world. Several studies have reported that restoration can recover much of the biodiversity and ES lost due to degradation [6]. On the other hand, studies have called into question the effectiveness of wetland restoration, suggesting that its positive impacts depend strongly on factors such as ecosystem type and restoration actions [5]. For example, some authors have suggested that current wetland restoration methods are too slow and incomplete to allow recovery of biological structure and biogeochemical function [7]. Therefore the effectiveness of wetland restoration remains controversial, and this is in part because different studies have applied different standards to evaluate outcomes [6]. At the same time, most studies evaluating wetland restoration, including a recent meta-analysis [7], have not directly assessed ES recovery or how well restoration methods work for diverse types of organisms.

Recovering biodiversity and recovering ES can be regarded as distinct goals of wetland restoration, with a given restoration focusing on one or the other. However, assessing both types of recovery simultaneously is important for several reasons. Biodiversity and ES of restored ecosystems often do not reach pre-degradation levels or the levels of similar natural ecosystems, and recovery of biodiversity may correlate with recovery of ES [8], [9]. Indeed, recovery of biodiversity may be a prerequisite for recovery of ES [7]; for instance, increasing biodiversity enhances key ES such as primary productivity [10] and soil erosion control [11]. Thus, comparable recovery of biodiversity and ES may indicate a win-win outcome for ecosystem and society alike. Additionally, assessments of wetland restoration should consider the context in which the restoration occurs, since restoration effectiveness may strongly depend on the type of ecosystem being restored, its pre-restoration condition, and the factors responsible for its degradation. By analyzing wetland restoration simultaneously in terms of biodiversity and ES, we can identify factors that affect the recovery of either or both, allowing us to develop recommendations for researchers and practitioners.

To develop an evidence-based approach for planning and assessing wetland restoration, we conducted a meta-analysis of the peer-reviewed literature to address the following four questions: (1) how much biodiversity and (2) how much of ES levels can be recovered through wetland restoration, (3) whether biodiversity and ES recovery correlate, and (4) whether the effectiveness of biodiversity and ES recovery depends on context, including ecosystem type, cause of degradation, restoration action, experimental design, and restoration age. In examining what the literature says on these questions, we hope to inform and improve efforts to restore the biodiversity and ES of degraded wetlands.

## Methods

### Literature search

We systematically searched the research literature to identify quantitative studies of the effects of ecological restoration on biodiversity and ES of non-marine aquatic and semi-aquatic degraded wetlands. We searched the ISI Web of Knowledge database (www.isiwebofknowledge.com), as it provides access to peer-reviewed studies. We searched studies published between 1970 and 2010 using the following string of search terms: (riparian OR river* OR lake OR mangroves OR marsh OR stream OR wetland) AND (restor* OR re-creat* OR rehabilitat* OR forest* OR reforest* OR afforest* OR plant* OR recover*) AND ((ecosystem OR environment) AND (service OR function*)). Preliminary search results were filtered to include only the following ISI-defined subject areas: “agriculture”, “biodiversity and conservation”, “environmental sciences and ecology”, “fisheries”, “forestry”, “marine and freshwater biology”, “plant sciences”, “water resources”, and “zoology”. This resulted in a list of 1,931 references.

For inclusion in our meta-analysis, studies had to focus on at least one estuarine, lacustrine, palustrine, or riverine wetland, as defined by [1], as well as report the following information:

1. Quantitative assessment of passive restoration (i.e. natural regeneration) or active restoration in terms of variables related to biodiversity and/or to the supply of one or more wetland ES (**Table 1**) consistent with the framework of the Millennium Ecosystem Assessment [4], according to which biodiversity underpins all ES;
2. Comparison of restored wetland with either degraded or natural wetland;
3. Sample size of the reported data and at least a variance estimate of such data.

A total of 70 studies (**Supporting information S1**) satisfied these criteria and were included in our meta-analysis. The number of observations included in each analysis is shown in the corresponding figures.

### Database building and effect size estimation

We constructed a computer database in which rows were observations and columns were properties of those observations (**Supporting information S2**; **Table S1**). For each study we extracted data on the variables used to measure the impacts of restoration (response variables). Separate databases were built for biodiversity and ES response variables. Whether we used one or the other database, or some combination of columns from both of them, depended on the specific question being addressed. Each measurement of restoration impact was recorded as a separate row in the database, even when the measurements came from the same study. Measurements were also recorded separately when the original study assumed spatially independent conditions within the same study site (e.g. measurements made near the shore vs. made on the open water of the same wetland).

We extracted data on type of wetland and ecosystem, the principal causes of degradation, specific restoration action(s) implemented, experimental design used to assess restoration outcomes, and the time elapsed since completion of the last restoration action (restoration age). All variables except restoration age were nominal and assigned to categories specifically created for our analyses (**Supporting information S3**).

Since our meta-analysis included studies differing considerably in response variables and experimental designs, we assessed the effects of restoration on biodiversity and ES relative to a control using response ratios (RRs) as the effect size metric. As an indicator of the outcome of restoration, we calculated RRs of the restored wetlands relative to reference natural wetlands [ln(Rest/Ref)] and to degraded wetlands [ln(Rest/Deg)] for each measure of the biodiversity and ES extracted from the studies. Most response variables were expected to correlate positively with biodiversity or a particular ES; for example, greater biomass was predicted to mean a higher level of supporting or provisioning ES. However, some response variables were predicted to correlate negatively with biodiversity or ES; for example, a greater concentration of a water or soil contaminant or a greater abundance of non-native species were predicted to reduce, respectively, provisioning ES and biodiversity. In these cases we inverted the sign of the RR (**Supporting information S2**).

We performed separate analyses to compare restored and degraded wetlands and to compare restored and natural wetlands [9] (**Supporting information S3**). RR calculations and statistical analyses were performed using MetaWin v2.1 [12].

### Biodiversity recovery

All possible measures of biodiversity for which the included studies reported data were used to calculate RRs; these measures included (a) species, gender, taxon or family richness; and (b) indices of species abundance, diversity, similarity, and composition. Using biodiversity measures calculated for different taxonomic levels or by different formulas enabled us to screen for differences in responses to restoration at different levels of ecological complexity [9], [13]. Each extracted datum was assigned to a single organism type. Data were analyzed using categorical, random-effects models because the data were most likely to satisfy the assumptions of these models [12]; the categories in the model were organism types.

To evaluate possible pseudo-replication effects, we calculated the mean RR for each of the three largest categories: macroinvertebrates, aquatic invertebrates, and vascular plants, using only one randomly selected effect size from each study. These mean RRs were similar to the means obtained when all effect sizes from each study were included, and the bias-corrected 95% bootstrap confidence interval of the reduced dataset overlapped with that of the entire dataset (**Table S2**). Therefore we retained all the data in our meta-analysis, similar to Rey Benayas *et al.* [9] and Vilà *et al.* [13].

### ES recovery

Response variables were related to a wide variety of ES, so multiple RR-ES combinations were included as separate rows in the database (**Table S1**). The parallel assessment of these multiple associations allowed us to capture the simultaneous supply of several ES [14], [15]. To avoid counting the same data more than once in a meta-analysis, we performed a separate meta-analysis for each ES using a random-effects model. We considered this approach suitable because we wanted to evaluate each ES separately, rather than the heterogeneity among different ES.

### Correlation between biodiversity and ES recovery

We assessed the correlation between biodiversity recovery and ES recovery using the Spearman rank coefficient to quantify the correlation between the corresponding RRs. We used only RRs from studies that evaluated both biodiversity and ES, and we treated each of these studies as an independent sample. When the same study reported multiple measures of biodiversity or ES, the related RRs were averaged to generate an overall RR for biodiversity and an overall RR for ES for each study, thereby minimizing the risk of pseudo-replication. This approach led us to combine the four major ES types in order to ensure adequate sample size [9].

### Context dependence of biodiversity and ES recovery

We used linear mixed-effects models to evaluate whether the effects of restoration on biodiversity and ES varied with context. Context was parameterized using four nominal fixed factors (ecosystem type, main cause of degradation, restoration action, and experimental design) and the continuous fixed factor of restoration age, defined as the decimal logarithm of the number of months between completion of the last restoration action and evaluation. We added a fifth nominal fixed factor with two levels (biodiversity or ES) because we used RRs for both biodiversity and ES recovery in the analysis. Study site was the random-effect factor and RR was the dependent variable.

We also built a second model in which we reduced the degrees of freedom by including only factor categories containing at least 30 observations. Since this reduced the average sample size in each category, we discarded this model in favor of the first. Finally, we applied a backward elimination procedure in which non-significant terms (*p*<0.05) were removed in order of decreasing *p* value. The selected final model contained main effects but no interactions. All model building and refinement was carried out using Data Desk v6 [16].

## Results

The 70 studies analyzed here were distributed across 62 locations in 14 countries (**Supporting information S4**). Riverine wetlands were the best-represented ecosystem type (38% of studies), followed by lacustrine wetlands (27%), and finally estuarine (18%) and palustrine wetlands (17%). Nearly all studies (68) were field-based comparisons, including three passive restoration studies (4%). The remaining two studies (3%) involved one field and one greenhouse experiment.

### Biodiversity recovery

**Figure 1:**
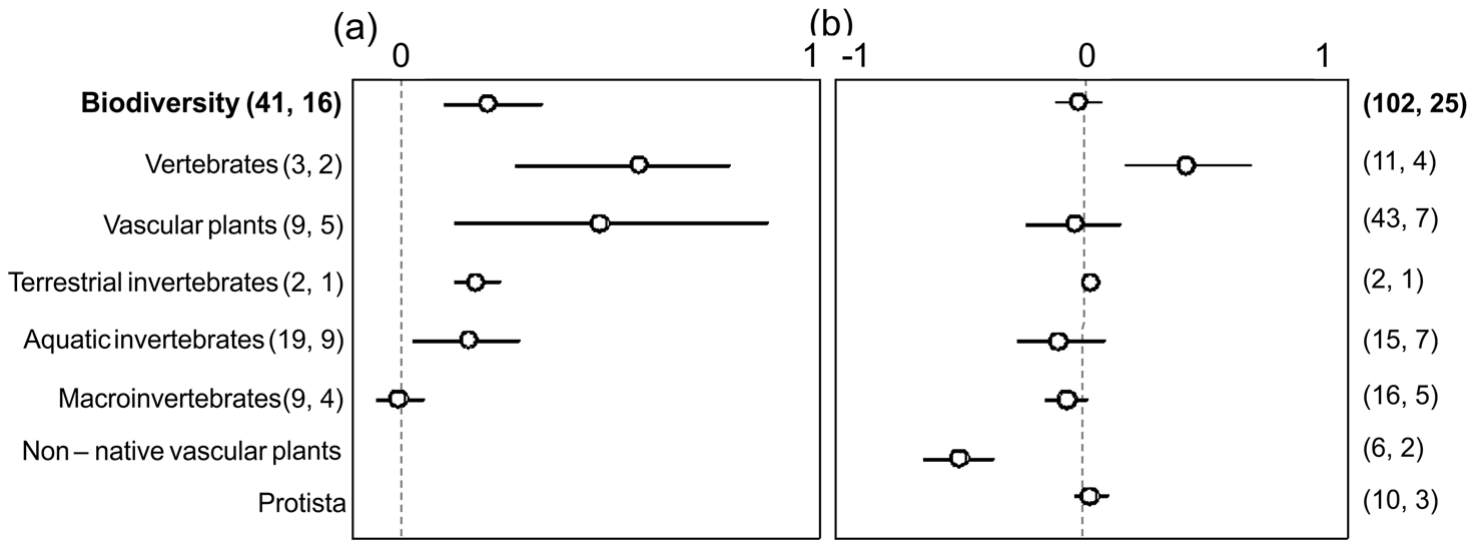
Mean effect size (response ratio) of ecological restoration on overall biodiversity and biodiversity of specific types of organisms in restored wetlands with respect to (a) degraded wetlands or (b) natural wetlands. Numbers in parentheses indicate the sample size (number of comparisons) followed by the numbers of studies. Bars extending from the means indicate bias-corrected 95% bootstrap confidence intervals. A mean effect size is significantly different from zero if the 95% confidence interval does not overlap with it. In comparison (a), no data were available on non-native vascular plants and protists. In comparison (b), the confidence interval for terrestrial invertebrates is not visible because it is smaller than the mean marker.

Restoring degraded wetlands enhanced biodiversity by 19% (**Fig. 1a**); and biodiversity in restored wetlands did not significantly differ from that in natural wetlands (**Fig. 1b**). Restoration significantly enhanced the diversity of vertebrates (+53%), vascular plants (+45%), and terrestrial (+17%) and aquatic (+15%) invertebrates, but it had no significant effect on macroinvertebrate diversity. Restored and natural wetlands showed similar diversity of vascular plants, aquatic invertebrates, macroinvertebrates and protists. In contrast, these two types of wetlands differed significantly in the diversity of non-native vascular plants, which was 44% lower in restored wetlands, and in vertebrate diversity, which was 37% higher in restored wetlands.

### ES recovery

**Figure 2:**
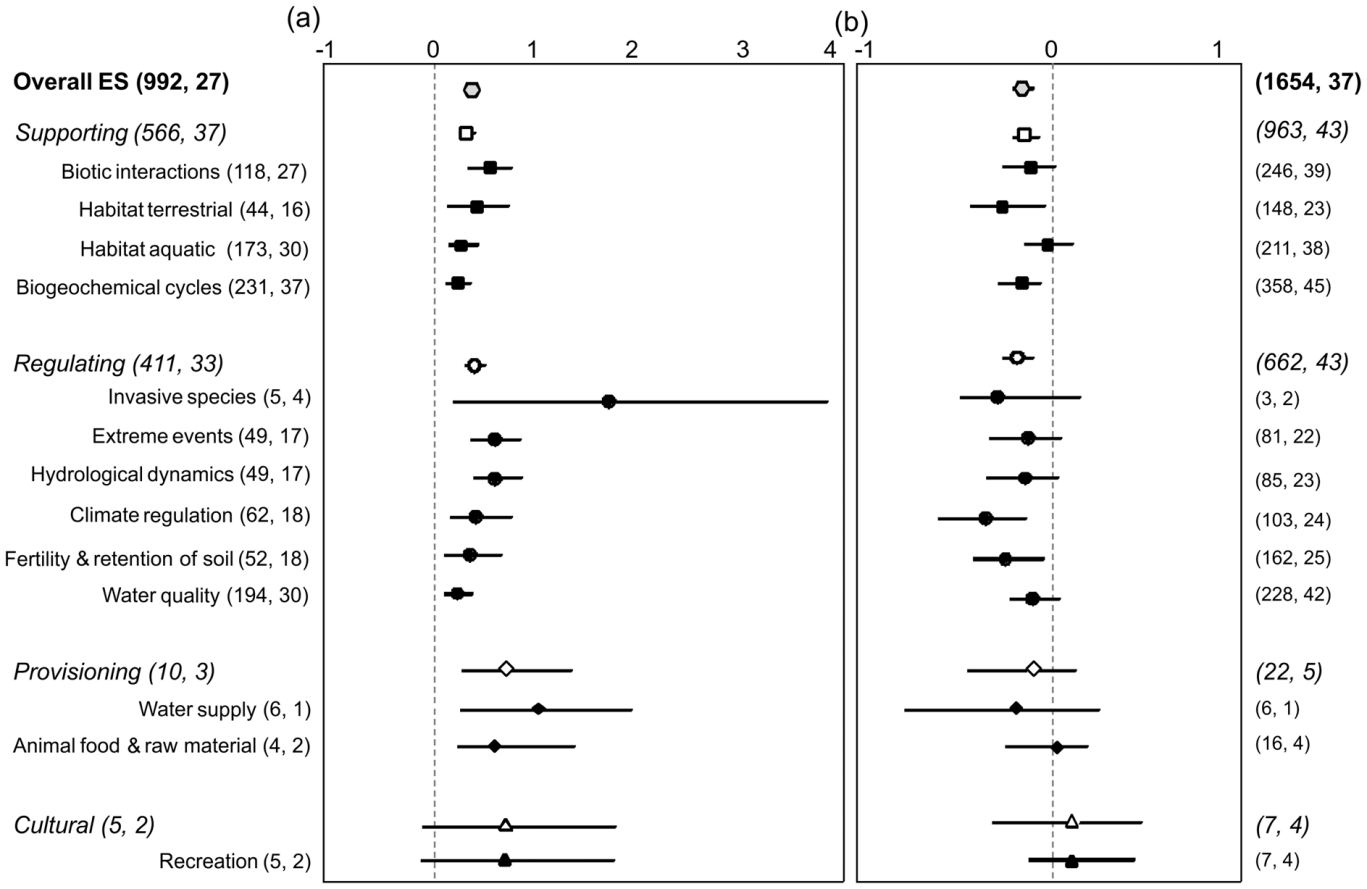
Mean effect size (response ratio) of ecological restoration on four major ES types defined by the MEA (2005) and on 13 individual ES (see details in **Table 1**) in restored wetlands with respect to (a) degraded wetlands or (b) natural wetlands. Bars extending from the means indicate bias-corrected 95% bootstrap confidence intervals. A mean effect size is significantly different from zero if the 95% confidence interval does not overlap with it. Numbers in parentheses indicate the sample size (number of comparisons) followed by the numbers of studies.

Overall ES supply was 43% higher in restored wetlands than in degraded ones (**Fig. 2a**), but 13% lower than in natural wetlands (**Fig. 2b**). Compared to degraded wetlands, restored wetlands showed much greater supply of provisioning ES (+80%), regulating ES (+47%) and supporting ES (+40%), while the two types of wetlands showed similar supply of cultural ES. Compared to natural wetlands, restored wetlands showed similar supply of provisioning and cultural ES, but lower supply of regulating (−22%) and supporting ES (−16%).

Restoration increased most individual ES that we examined, although not to the same extent (**Fig. 2a**). Restoration increased the supply of supporting services, with increases ranging from 32% for biogeochemical cycling to 61% for biotic interactions. Increases in the supply of regulating services ranged from 31% for water quality to 176% for invasive species control. Restoration also increased both provisioning services examined in our meta-analysis: water supply (+108%) and the supply of food or raw materials of animal origin (+65%). For most individual ES that we examined, restored and natural wetlands tended to supply similar amounts (**Fig. 2b**). Exceptions, in decreasing order of difference between the two wetland types, were climate regulation, the supply of which was −30% lower in restored wetlands; provision of terrestrial habitat, −22%; regulation of fertility and soil erosion, −21%; and biogeochemical cycles, −14%.

### Correlation between biodiversity and ES recovery

**Figure 3:**
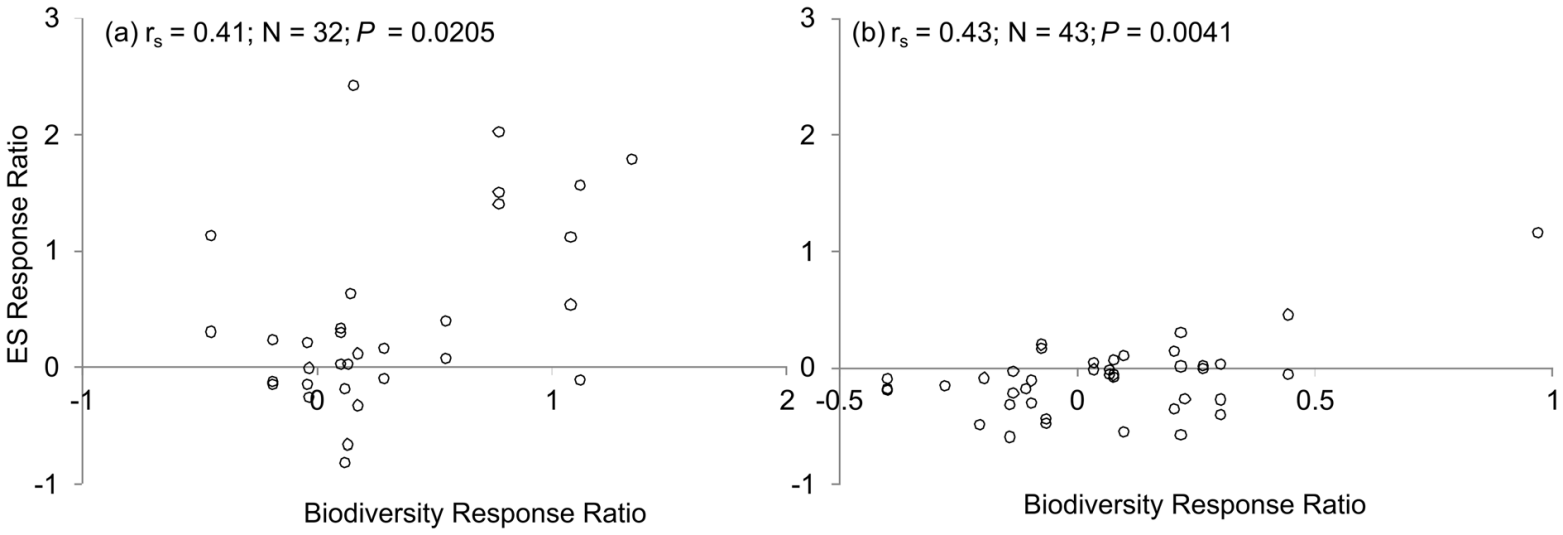
Spearman rank correlations between biodiversity and ES supply in restored wetlands with respect to (a) degraded wetlands or (b) natural wetlands.

Biodiversity and ES response ratios positively correlated in comparisons of restored and degraded wetlands (**Fig. 3a**) and in comparisons of restored and natural wetlands (**Fig. 3b**).

### Context dependence of biodiversity and ES recovery: restored vs. degraded wetlands

Comparison of restored and degraded wetlands showed that restoration effects depended on the following factors, listed in order of decreasing importance: main cause of degradation, restoration action, experimental design, and ecosystem type (**Table 2**). In contrast, restoration age did not significantly affect restoration outcomes. These results were the same for the two outcomes of biodiversity recovery and ES recovery.

**Table 2:** Results of mixed linear models assessing the influence of ecological context factors on the effects of restoration on biodiversity and ecosystem services of wetlands.

Factor	Wetland comparison					
	*Restored vs. Degraded*			*Restored vs. Natural*		
	F	*P*	Explained variance (%)	F	*P*	Explained variance (%)
ResAct	5.32_9,300_	<0.0001	11.7	2.35_9,506_	0.0133	3.8
DegFac	6.03_4,300_	0.0001	5.9			
EcoType	2.82_8,300_	0.0051	5.5			
ExpDes	5.24_2,300_	0.006	2.6			
B/ES				3.93_3,506_	0.0038	2.8

*Abbreviations:* B/ES, ratio of biodiversity to ecosystem services; DegFac, degrading factor; EcoType, ecosystem type; ExpDes, experimental design; ResAct: restoration action.

Context variables explained relatively little variance (25.7%) in biodiversity and ES recovery. Nevertheless, the improvement in biodiversity and ES due to restoration varied substantially for different wetland types: salt marshes (+104%), freshwater marshes (+73%), rivers (+100%), lakes (+45%), mangroves (+33%), and streams (+9%; **Fig. S1**).

Restoration significantly ameliorated all causes of degradation that we examined, except for the presence of invasive species (**Fig. S2**). Seven of the 10 restoration actions reported by the included studies showed significant effects on biodiversity and ES supply (**Fig. S3**), with habitat creation leading to the greatest benefit (+119%), followed by soil amendment and revegetation (+91%), and passive restoration in third place (+57%). Of all restoration actions examined, exotic species removal was associated with the lowest effect size, which did not achieve statistical significance. Restoration showed significant positive effects on biodiversity and ES recovery for the three types of experimental designs in the included studies: paired experiments (+61%), before-after experiments (+33%) and control-impact experiments (+22%; **Fig. S4**).

### Context dependence of biodiversity and ES recovery: restored vs. natural wetlands

Comparison of restored and natural wetlands showed that restoration significantly improved recovery of biodiversity and ES supply (**Table 2**), although as before, the final model explained only a fraction of the variance (15.2%). All restoration actions led to full recovery of biodiversity and ES supply except for soil amendment and revegetation, which led to −124% lower levels of biodiversity and ES supply than in natural wetlands; passive restoration, which led to −31% lower levels; manipulation of structural heterogeneity, −15%; and hydrological dynamics, −21% (**Fig. S3**).

## Discussion

### Biodiversity recovery

Our global meta-analysis, including70 studies conducted in 14 countries, shows that wetland restoration increased biodiversity in degraded wetlands, consistent with another global meta-analysis of different ecosystem types [9]. In fact, restoration increased the biodiversity of native organisms to levels similar to those in natural wetlands. To be sure, restoration did not improve biodiversity of all organisms uniformly. Restoration increased vertebrate diversity to levels above those in natural wetlands, though this result may only be transient, since vertebrate richness can vary substantially over time [17]. Conversely, restoration led to levels of biodiversity of non-native vascular plants lower than levels in natural wetlands. Both of these outcomes may reflect the large, persistent effects of exotic plants on the habitat structure, biodiversity and functioning of wetlands [5]. In addition, wetlands dominated by exotic, invasive plants tend to support fewer native animal species and more invasive animals [5].

Greater diversity by itself is insufficient to ensure high ecosystem functioning [18]. Potentially even more important are the identities and relative proportions of species involved in the restoration process, as well as their ecological and functional properties. Unfortunately, most studies in our meta-analysis reported aggregate measures of richness or diversity but not community composition (**Supporting information S1**). Indeed a previous meta-analysis of how restoration affects major groups of organisms was restricted to calculating aggregate results for three general categories of vertebrates, macroinvertebrates, and plants [7]. Higher taxonomic and functional resolution is needed to explore the potentially quite different effects of restoration on organisms that can differ even within a class like vertebrates. Therefore, restoration studies dealing with species composition, community structure and functional ecology are urgently needed.

### ES recovery

Our meta-analysis showed that restoration enhanced ES supply in degraded wetlands. The results also showed that it is more difficult to recover ES supply than to recover biodiversity; an alternative or complementary interpretation is that full recovery of ES supply takes longer than full recovery of biodiversity. Either interpretation is consistent with the meta-analysis by Rey Benayas *et al.* [9], but inconsistent with the analysis of North American wetlands by Dodds *et al.* [8].

Restoration did not enhance ES uniformly across all individual ES examined. We observed that restored wetlands provided, on average, 36% higher levels of provisioning, regulating and supporting ES than did degraded wetlands, but similar levels of cultural services. To be sure, we did not expect uniform recovery of all individual ES, given the heterogeneity of ES and wetland types included in the meta-analysis; wetlands types are known to differ in ecological dynamics, recovery rates and extents of recovery [7].

Our finding that restoration increased supply of provisioning services more than the supply of other ES may reflect the fact that, among the included studies, the desired outcomes when restoring provisioning services (e.g. abundance of target species) were generally better defined and more homogeneous than were objectives for regulating, supporting, and cultural services. Effect sizes for these last three services showed wide confidence intervals in our study, suggesting higher intra-class heterogeneity than effect sizes for provisioning services [12]. Small sample size may explain our finding that restoration did not significantly affect cultural services. Compared to natural wetlands, restored wetlands showed similar supply of provisioning and cultural services but lower supply of regulating services (mainly climate regulation, soil fertility and erosion) and supporting services (mainly biogeochemical cycles and provision of terrestrial habitat). The lower levels of climate and soil regulation, biological structure and biogeochemical cycles may reflect the intrinsically slow recovery rates reported for these surrogate variables [7]. In contrast, faster recovery rates have been reported for the water regulation variables in our study, such as hydrological dynamics and water quality, and these latter variables indeed showed full recovery.

Analysis of the ES database, which included abundance data on both non-native plant and animal species, showed that restoration increased regulation of non-native species by reducing their abundance. This result is different than our finding that restoration increased the diversity of such species, though it should be noted that the biodiversity database contained data on non-native plants but not non-native animals. The abundance of non-native species may decrease rapidly during the restoration process because these species are directly eradicated. However, a reduction in abundance, which reduces the supply of ES, does not necessarily indicate a decrease in species diversity, such as when a habitat contains several rare species in low abundance. Thus, assessment of restoration should take into account both abundance and diversity indicators.

### Correlation of biodiversity and ES recovery

The relationship between biodiversity and ES supply remains poorly understood [19], yet it is crucial to work out because it has significant implications not only for restoration science but also for wider society, economics, and policy [20], [21]. Our results showed that changes in biodiversity positively correlated with changes in ES supply in a variety of wetlands, ecosystem types and scales, which supports a functional role for biodiversity in the supply of ES [7], [9]. This positive relationship is good news for restoration efforts, as it demonstrates the possibility of win-win scenarios for restoring biodiversity and ES. However, such win-win gains have not always proven feasible in practice, especially in restoration projects involving geographically dispersed areas [22]. Future research should explore how to optimize the synergy between biodiversity and ES supply in the design of management and conservation programs involving restoration.

The relationship between biodiversity and ES is also important because it has consequences beyond ecosystem restoration. For example, increasing plant diversity has been shown to enhance the provision of goods from plants and the regulation of erosion, invasive species and pathogens [23]; thus, recovering plant diversity may contribute to the recovery of ES beyond the immediate effects of restoration activities. Future research is needed to disentangle direct and indirect effects of restoration on biodiversity and ES, as well as clarify how the two types of effects interact.

### Context dependence

Our meta-analysis identified several context factors that significantly affected biodiversity and ES recovery in restored wetlands, including ecosystem type, main cause of degradation, restoration action taken, and experimental design used to assess the restoration. This highlights the need to take context into account when evaluating the effects of wetland restoration. Particularly, examining interaction effects may generate useful insights, but the risk of multiple interactions, including two or even three factors, is too high for the relatively low statistical power of our model.

Our results also showed that biodiversity and ES recovery did not depend on restoration age. Nevertheless, they may depend on how long the restoration process took, on how many times a restoration action was repeated and on the conditions of the degraded wetland prior to restoration. Unfortunately most of the studies included in our meta-analysis did not report such data. The type and duration of interventions required in restoration depend heavily on the type and extent of ecosystem damage [24]. Future research should examine these context factors in greater detail.

Our finding that restoration effects depended on ecosystem type is consistent with an earlier meta-analysis showing that wetlands with more hydrologic flow exchange recovered faster than those that did not receive external water flow [7]. We obtained different results showing that outcomes of restoration were unrelated to flow exchange, e.g. biodiversity and ES in rivers and streams were enhanced in very different amounts. Despite these differences, the available evidence strongly indicates that the effectiveness of restoration is habitat-specific, arguing for the need for more research into how to tailor restoration projects to particular environments and how to assess their outcomes accordingly [6].

Our meta-analysis showed that only restoration action determined how close the biodiversity and ES supply of restored wetlands approached those of natural wetlands. This finding implies that unless the correct restoration action is chosen from the beginning, which is often impossible, the restored wetland may not come as close as possible to natural conditions. Applying a combination of restoration actions may therefore improve the likelihood of success.

Taken together, the results of our mixed models suggest that comparisons of degraded, restored, and reference conditions should be carried out to guide and evaluate restoration based on multiple indicators of both biodiversity and ES. These indicators should be consistent with the specific restoration goals [25], which can vary greatly depending on the context and project [26]. Our models further suggest that restoration programs should involve multiple actions to improve the likelihood of success.

### Implications for wetland restoration

Comparing degraded, restored and reference conditions to guide restoration may not be feasible in many cases because the irreversibility of much of man-made ecosystem damage makes it difficult to simulate the pre-degradation condition accurately [27], and because movement of restored wetlands away from reference conditions makes it difficult to project desired outcomes [7], but it should be advisable. This highlights the need for designing restoration programs with multiple, alternative goals in mind [27], [28]. These goals should take into account the social context and human values associated with decisions about wetland management and restoration. The concept of ES can be a robust guide for wetland restoration decision-making because it identifies and quantifies valuable goods and describes the processes and components that provide essential services [29]. Since several ES are difficult to measure directly, surrogate measures of ecosystem function can be used instead [30].

Accurately assessing the impact of restoration on biodiversity and ES supply requires identifying the particular ecosystem attributes in need of restoration. To capture potential differences in the restoration of individual ES, we linked the response variables to ES based on specific measures routinely included in ecological studies [31]. In addition, we evaluated the effects of response variables on multiple ES, since the variables may have indirect or unclear links to several ES that significantly affect restoration outcomes. For instance, although all plant species capture carbon, thereby increasing the supply of one ES, non-native species may have detrimental effects on other ES such as biotic interactions. A single restoration action may simultaneously affect various ES or act synergistically as a ‘cascade’ across trophic levels [14]. A restoration action may enhance the supply of one ES while precluding the supply of another [32], or it may generate a disservice, such as the release of greenhouse gases. Therefore, analyses of restoration data should assess both the direction and magnitude of associations between response variables and individual ES [14]. Taking into account the multiple ES associated with a restoration action facilitates the identification of tradeoffs or compromises when planning wetland restoration in which the overriding goal is optimizing multiple ES [29].

Cost plays an important role in restoration planning because it may limit the desired outcomes [33], [34]. Surprisingly, the studies included in our meta-analysis did not address the issue of restoration costs. Costs are an important factor not only during restoration but also after: monitoring of wetlands following their restoration, mitigation or creation is often too brief because it is expensive to evaluate all the ecosystem functions involved.

These elements define a complex scenario for decision makers. Key to guiding decisions will be a systematic account of the relationships between wetland restoration variables and the supply of individual ES, for which the evidence base needs to be expanded. Indeed the low positive correlation between the recovery of biodiversity and ES suggests that reliable modeling of restoration outcomes will require incorporating multiple indicators that capture biodiversity, ES supply, and ecosystem processes. Such indicators should also include performance indicators that describe how much of available ES can be exploited [19], since biodiversity-related ES, for example, vary over time and space and are species-dependent. This poses a challenge for model-building, since simple models for simultaneously maximizing biodiversity and ES are unrealistic or ambitious [35], such that the two variables are not necessarily maximized in the same wetland [6]. The model that we have developed here may provide a basis for future studies that optimize biodiversity and ES supply for specific habitats and contexts.

## Conclusions

Our meta-analysis strongly supports the idea that ecological restoration increases both biodiversity and ES supply in degraded wetlands, thereby benefiting the human communities that interact with and depend on them. The detailed effects of restoration depend heavily on context factors, emphasizing the need for habitat-specific planning and assessment of restorations [6]. Questions posed years ago remain largely unanswered today, such as “To what extent and over what time scale can ES be restored? [36] and “To what extent can mankind substitute for ES?” [37]. While restoration ecology is not obliged to answer these questions, exploring them may help improve the flows of ES and improve human well-being. Addressing these questions will require deepening our understanding of the links between restoration actions and changes in biophysical and ecological processes that generate ES [30]. While such research should inform and improve growing efforts to restore and mitigate loss of wetland area and loss of wetland ecosystem functions [35], they should not take importance away from efforts to conserve natural wetlands and avoid environmental degradation in the first place [8], [9].

## Supporting Information

Checklist S1 (DOC)

Supporting information S1 Studies used in the meta-analysis.
(DOC)

Supporting information S2 Additional information on database building.
(DOC)

Supporting information S3 Methodological details of the meta-analysis.
(DOC)

Supporting information S4 Overview of studies included in the meta-analysis.
(DOC)

Figure S1 **Restoration effects by ecosystem type.**
(TIFF)

Figure S2 **Restoration effects by main degrading factor.**
(TIFF)

Figure S3 **Restoration effects by restoration action.**
(TIFF)

Figure S4 **Restoration effects by experimental design.**
(TIFF)

Figure S5 **PRISMA 2009 Flow Diagram.**
(DOC)

Table S1 Linked databases used in the meta-analysis of biodiversity and ES.
(XLS)

Table S2 Comparison of biodiversity meta-analyses using a reduced or complete database.
(DOC)
