# Supplementary material for: Restoration Enhances Wetland Biodiversity and Ecosystem Service Supply, but Results Are Context-Dependent: A Meta-Analysis
Source: PLoS One. 2014 Apr 17;9(4):e93507. doi: 10.1371/journal.pone.0093507 (PMC3990551; doi:10.1371/journal.pone.0093507)
Supplement: Supporting information S1 — Studies used in the meta-analysis. (DOC) [file pone.0093507.s002.doc]

**SUPPORTING INFORMATION S1.** List of studies included in the meta-analysis. The studies are numbered according to their **R ID** (see Appendix S2).

1. Katz, G.L., Stromberg, J.C. & Denslow, M.W. (2009). Streamside herbaceous vegetation response to hydrologic restoration on the San Pedro River, Arizona. *Ecohydrol*, 2, 213-225.
2. Kovalenko, K., Dibble, E.D. & Fugi, R. (2009). Fish feeding in changing habitats: effects of invasive macrophyte control and habitat complex. *Ecol. of Freshwater Fisheries*, 18, 305-313.
3. Sutton-Grier, A.E., Ho, M. & Richardson, C.J. (2009). Organic amendments improve soil conditions and denitrification in a restored riparian wetland. Wetlands, 29, 343-352.
4. Zacheis, A. & Doran, K. (2009). Resistance and resilience of floating mat fens in interior Alaska following airboat disturbance. Wetlands, 29, 236-247.
5. Aldridge, K.T., Brookes, J.D. & Ganf, G.G. (2009).Rehabilitation of Stream Ecosystem Functions through the Reintroduction of Coarse Particulate Organic Matter. Rest. Ecol., 17, 1, 97-106.
6. Entrekin, S.A., Tank, J.L., Rossi-Marshall, E.J., Hoellein, T.J. & Lamberti, G.A. (2008). Responses in organic matter accumulation and processing to an experimental wood addition in three headwater streams. Freshwater Biol., 53, 1642-1657.
7. Hoeltje, S.M. & Cole, C.A. (2009). Comparison of function of created wetlands of two age classes in central Pennsylvania. Environm. Manage., 43, 597-608.
8. Sheley, R.L., Laufenberg, S.M., Jacobs, J.S. & Borkowski, J. (2007). Restoring species richness and diversity in a Russian Knapweed (*Acroptilon repens*) infested riparian plant community using herbicides. Weed Science, 55, 331-318.
9. Walton, M.E., Le Vay, L., Lebata, J.H., Binas, J. & Primavera, J.H. (2007). Assessment of the effectiveness of mangrove rehabilitation using exploited and non-exploited indicator species. Biol. Con., 138, 180-188.
10. Meyer, C.K., Baer, S.G. & Whiles, M.R. (2008). Ecosystem recovery across a chronosequence of restored wetlands in the Platte River Valley. Ecosys., 11, 193-208.
11. Li, X., Liquan, Z. & Zhang, Z. (2006). Soil bioengineering and the ecological restoration of riverbanks at the Airport Town, Shangai, China. Ecol. Eng., 26, 304-314.
12. Jian, J. G. & Shen, Y. F. (2007). Studies on the restoration succession of PFU microbial communities in a pilot-scale mesocosm. Chemosphere, 68, 637-646.
13. Lester, R.E., Wright, W. & Jones-Lennon, M. (2007). Does adding wood to agricultural streams enhance biodiversity? An experimental approach. Marine and Freshwater Research, 58, 687-698.
14. Xu, H., Ye, M., Song, Y. & Chen, Y. (2007). The natural vegetation responses to the groundwater change resulting from water conveyances to the lower Tarim River. Environmental Monitoring Assessment, 131, 37-48.
15. Ballantine, K. & Schneider, R. (2009). Fifty-five years of soil development in restored freshwater depressional wetlands. Ecol. Appl., 19, 1467-1480.
16. Crawford E.R., Day, F.P. & Atkinson, R.B. (2007). Influence of environment and substrate quality on root decomposition in naturally regenerating and restored Atlantic White Cedar wetlands. Wetlands, 27, 1.
17. Ström, L., Lamppa, A. & Christensen, T.R. (2007). Greenhouse gas emission from a constructed wetland in southern Sweden. Wetlands Ecol. and Manage., 15, 43-50.
18. Spieles, D.J., Coneybeer, M. & Horn, J. (2006). Community structure and quality after 10 years in two central Ohio mitigation bank wetlands. Environ. Manage., 38, 837-852.
19. Smith, J.M., Castro, H. & Ogram, A. (2007). Structure and function of methanogenes along a short-term restoration chronosequence in the Florida Everglades. Applied and Environ. Microbiol., 73, 4135-4141.
20. Mckie, B.G., Petrin, Z. & Malmqvist, B. (2006). Mitigation or disturbance? Effects of liming on macroinvertebrate assemblage structure and leaf litter decomposition in the humic streams of northern Sweden. J. of Appl. Ecol., 43, 780-791.
21. Pegg, M.A., Irons, K.S., O'Hara, T.M. & McClelland, M.A. (2006). Initial response of a floodplain lake fish community to water-level stabilization. Ecol. of Freshwater Fish, 15, 40-47.
22. Lovelock, C.E., Feller, I.C., Ellis, J., Schwartz, A.M., Hancock, N., Nichols, P. & Sorrell, B. (2007). Mangrove growth in New Zealand estuaries; the role of nutrient enrichment at sites with contrasting rates of sedimentation. Oecol, 153, 633-641.
23. Jackson, R.D., Allen-Díaz, B., Oates, L.G. & Tate, K.W. (2006). Spring-water nitrate increased with removal of livestock grazing in a California oak savanna. Ecosys., 9, 254-267.
24. Diemont, S.A.W. (2006). Mosquito larvae density and pollutant removal in tropical wetland treatment systems in Honduras. Environ. Intern, 32, 332-341.
25. Casper, A.F., Thorp, J.H., Davies, S.P. & Courtemanch, D.L. (2006). Ecological responses of zoobenthos to dam removal on the Kennebec River, Maine, USA. Large Rivers, 16, 541-555.
26. Bosire, J.O., Dahdouh-Guebas, F., Wartel, J.G., Kazungu, J. & Koedam, N. (2006). Success rates of recruited tree species and their contribution to the structural development of reforested mangrove stands. Mar. Ecol. Progr. Series, 325, 85-91.
27. Borders, B.D., Pushnik, J.C. & Wood, D.M. (2006). Comparison of leaf litter decomposition rates in restored and mature riparian forest on the Sacramento River. Res. Ecol., 14, 308-315.
28. Kinzie III, R.A., Chong, C., Devrell, J., Lindstrom, D. & Wolff R. (2006). Effects of water removal on a Hawaiian stream ecosystem. Pacific Sci., 60, 1, 01/47.
29. Lepori, F., Palm, D. & Malmqvist, B. (2005a). Effects of stream restoration on ecosystem functioning: detritus retentiveness and decomposition. J. of Appl. Ecol., 42, 228-238.
30. Greenfield, B.K., Siemering, G.S., Andrews, J.C., Rajan, M., Andrews Jr, S.P. & Spencer D.F. (2007). Mechanical shredding of water hyacinth (*Eichornia crassipes*): Effects on water quality in the Sacramento - San Joaquin River Delta, California. Estuaries and Coasts, 304, 627-640.
31. Greathouse, E.A., March, J.G. & Pringle, C.M. (2005). Recovery of a tropical stream after a harvest-related chlorine poisoning event. Freshwat. Biol., 50, 603-615.
32. Hein, T., Baranyi, C., Reckendorfer, W. & Schiemer, F. (2004). The impact of surface water exchange on the nutrient and particle dynamics in side-arms along the River Danube. Austria Sci. of the Tot. Environ., 328, 207-218.
33. Mayer, P.M., Megard, R.O. & Galatowitsch, S.M. (2004). Plankton respiration and biomass as functional indicators of recovery in restores wetlands. Ecol. Indic., 4, 245-253.
34. Parkyn, S.M., Davies-Colley, R.J., Halliday, N.J., Costley, K.J. & Croker, G.F. (2003). Planted riparian buffer zones in New Zealand: do they live up to expectations?. Res. Ecol., 11, 436-447.
35. Mayer, P.M. & Galatowitsch, S.M. (1999). Diatom communities as ecological indicators of recovery in restored prairie wetlands . Wetlands, 19, 765-774.
36. Lepori, F., Palm, D. & Malmqvist, B. (2005b). Does restoration of structural heterogeneity in streams enhance fish and macroinvertebrates diversity? Ecol. Appl., 15, 2060-2071.
37. Brooks, S.S., Palmer, M.A., Cardinale, B.J., Swan, C.M. & Ribblett, S. (2002). Assessing stream ecosystem rehabilitation: Limitations of community structure data. Res. Ecol., 10, 156-168.
38. Bosire, J.O., Dahdouh-Guebas, F., Kairo, J.G., Cannicci, S. & Koedam, N. (2004). Spatial variations in macrobenthic fauna recolonisation in a tropical mangrove bay. Biod. & Cons., 13, 1059-1074.
39. Andersen, D.C. & Nelson, S.M. (1999). Rodent use of anthropogenic and natural desert riparian habitat, lower Colorado River, Arizona. Regul. Rivers: Research & Manage., 15, 377-393.
40. Craft, C., Reader, J., Sacco, J.N. & Broome, S.W. (1999). Twenty-five of ecosystem development of constructed *Spartina alterniflora* (Loisel) marshes. Ecol. Appl., 9, 1405-1419.
41. McKenna, J.E. (2003). Community metabolism during early development of a restored wetland. Wetlands, 23, 35-50.
42. Matthews, J.W., Spyreas, G. & Endress, A.G. (2009). Trajectories of vegetation-based indicators used to assess wetland restoration progress. Ecol. Appl. , 19, 2093-2107.
43. La Peyre, M.K., Gossman, B. & Nyman, J.A. (2007). Assessing functional equivalency of nekton habitat in enhanced habitats: comparison of terraced and unterraced marsh ponds. Estuar. & Coasts, 30, 526-536.
44. Phillips, I.D., Vinebrook, R.D. & Turner, M.A. (2009). Experimental reintroduction of the crayfish species *Orconectes virilis* into formerly acidified Lake 302S (Experimental Lakes Area, Canada).Can. J. of Fish. & Aq. Sci., 66, 1892-1902.
45. Entrekin, S.A., Tank, J.L., Rossi-Marshall, E.J., Hoellein, T.J. & Lamberti, G.A. (2009). Response of secondary production by macroinvertebrates to large wood addition in three Michigan streams. Freshwat. Biol., 54, 1741-1758.
46. Guo, Q., Feng, Q. & Li, J. (2009). Environmental changes after ecological water conveyance in the lower reaches of Heihe River, northwest China. Environ. Geol., 58, 1387-1396.
47. Cronin, G., Lewis Jr., W.M. & Schiehser, M.A. (2006). Influence of freshwater on the littoral ecosystem structure and function of a young Colorado reservoir. Aq. Bot., 85, 37-43.
48. Roman, C.T., Raposa, K.B., Adamowicz, S.C., James-Pirri, M.J. & Catena, J.G. (2002). Quantifying vegetation and nekton response to tidal restoration of a New England salt marsh. Res. Ecol., 10, 450-460.
49. Petranka, J.W., Murray, S.S. & Kennedy, C.A. (2003a). Responses of amphibians to restoration of a southern Appalachian wetland: Perturbations confound post-restoration assessment. Wetlands, 23, 278-290.
50. Petranka, J.W., Kennedy, C.A. & Murray, S.S. (2003b). Responses of amphibians to restoration of a southern Appalachian wetland: A long-term analysis of community dynamics. Wetlands, 23, 1030-1042.
51. Levin, L.A. & Talley, T.S. (2002). Natural and manipulated sources of heterogeneity controlling early faunal development of a salt marsh. Ecol. Appl., 12, 1785-1802:
52. La Peyre, M.K., Gossman, B. & Piazza, B.P. (2009). Short- and Long-term response of deteriorating brackish marshes and open-water ponds to sediment enhancement by thin-layer dredge disposal. Estuar. & Coasts, 32, 390-402.
53. Hogan, D.M., Jordan, T.E. & Walbridge, M.R. (2004). Phosphorus retention and soil organic carbon in restored and natural freshwater wetlands . Wetlands, 24, 573-585.
54. Harig, A.L. & Brain, M.B. (1998). Defining and restoring biological integrity in wilderness lakes. Ecol. Appl., 81, 71-87.
55. Gratton, C. & Denno, R.F. (2005). Restoration of arthropod assemblages in a *Spartina* salt marsh following removal of the invasive plant *Phragmites australis*. Res. Ecol., 13, 358-372.
56. Graham, S.A., Craft, C.B., McCormick, P.V. & Aldous, A. (2005). Forms and accumulation of soil P in natural and recently restored peatlands-upper Klamath Lake, Oregon, USA. Wetlands, 25, 594-606.
57. Fennessy, M.S., Rokosch, A. & Mack, J.J. (2008). Patterns of plant decomposition and nutrient cycling in natural created wetlands. Wetlands, 28, 300-310.
58. Buchsbaum, R.N., Catena, J., Hutchins, E. & James-Pirri, M.J. (2006). Changes in salt marsh vegetation, *Phragmites australis*, and nekton response to increased tidal flushing in a New England salt marsh. Wetlands, 26, 544-557.
59. Edwards, K.R. & Kaili M. (2005). Aboveground and belowground productivity of *Spartina alterniflora* (Smooth cordgrass) in natural and created Lousiana salt marsh. Estuaries, 28, 252-265.
60. Ellis, L.M., Molles Jr., M.C. & Crawford, C.S. (1999). Influence of experimental flooding on litter dynamics in a Rio Grande riparian forest, New Mexico. Res. Ecol., 7, 193-204.
61. Craf, C., Megoninal, P., Broome, S., Stevenson, J., Freese, R., Cornell, J., Zheing, L. & Sacco, J. (2003). The pace of ecosystem development of constructed *Spartina alterniflora* marshes. Ecol. Appl., 13, 1417-1432.
62. Callaway, J.C., Sullivan, G., Zedler, J.B. (2003). Species-rich plantations increase biomass and nitrogen accumulation in a wetland restoration experiment. Ecol. Appl., 13, 1626-1639.
63. Meuleman, A.F.M., Beekman, J.Ph. & Verhoeven, J.T.A (2002). Nutrient retention and nutrient-use efficiency in *Phragmites australis* stands after wastewater application. Wetlands, 22, 712-721.
64. Meyer, C.M. & Whiles, M.R. (2008). Macroinvertebrate communities in restored and natural Platte River slough wetlands. J. of the North Am. Benthol. Soc., 27, 626-639.
65. Middleton, B., Devlin, D., Proffitt, E., McKee, K. & Foster Cretini, K. (2008). Characteristics of mangrove swamps managed for mosquito control in eastern Florida, USA. Marine Ecol. Progr. Series, 371, 117-129.
66. Meyer, C.M., Whiles, M.R. & Baer, S.G. (2010). Plant community recovery following restoration in temporally variable riparian wetlands. Res. Ecol., 18, 52-64.
67. Hill, M.T. & Platts, W.S. 1998). Ecosystem Restoration: A case study in the Owens River George, California. Fish. Hab., 23, 18-27.
68. Mayence, C.E., Marshall, D.J. & Godfree, R.C. (2010). Hydrologic and mechanical control for an invasive wetland plant, *Juncus ingens*, and implications for rehabilitating and managing Murray River floodplain wetlands, Australia. Wetl. Ecol. & Manage., 18, 717-730.
69. Carreira, J.A., Viñegla, B., García-Ruiz, R., Ochoa, V. & Hinojosa, M.B. (2008). Recovery of biochemical functionally in polluted flood-plain soils: The role of microhabitat differentiation through revegetation and rehabilitation of the river dynamics. Soil Biol. & Biochem., 40, 2088-2097.
70. Wong, S.W., Barry, M. J. Aldous, A.R., Rudd, N.T., Hendrixson, B. & Doehring, C.M. (2011). Nutrient release from a recently flooded delta wetland: Comparison of field measurements to laboratory results. Wetlands.
