# Supplementary material for: Restoration Enhances Wetland Biodiversity and Ecosystem Service Supply, but Results Are Context-Dependent: A Meta-Analysis
Source: PLoS One. 2014 Apr 17;9(4):e93507. doi: 10.1371/journal.pone.0093507 (PMC3990551; doi:10.1371/journal.pone.0093507)
Supplement: Supporting information S2 — Additional information on database building. (DOC) [file pone.0093507.s003.doc]

**SUPPORTING INFORMATION S2.** Additional information on database building.

Column headings and descriptions

**D ID:** Unique number assigned to each observation or measurement in each study.

**R ID**: Unique number assigned to each study.

**L ID:** Unique number (preceded by “L”) referring to the location for a single-site study or to each location within a multi-site study.

**ExpDes:** Type of experimental design (see "Database criteria" below).

**EcoType:** Type of ecosystem. Possible values: delta, lake, mangrove, freshwater marsh, salt marsh, riparian ecosystem, river, stream, wet meadow, or wetland. An ecosystem was defined as "wetland" by default if the study did not specify another type.

**WetlType:** Type of wetland, as defined by Ramsar (2006): riverine, palustrine, lacustrine, estuarine.

**CauDeg:** Causes of degradation in the study site (see "Database criteria").

**ResAct:** Specific restoration action applied. Passive restoration or elimination of the cause of degradation was included as a possible value.

**RA Group:** Overall category of restoration actions (10 categories total; see next "Database criteria").

**AgeRes:** Time interval between the completion of the last restoration action and measurement of restoration effects (see "Database criteria").

**VarMeas:** Description of the response variable measured.

**Biod:** Biodiversity.

**PF/RM:** Plant food / Raw material.

**AF/RM:** Animal food / Raw material.

**WS:** Water supply.

**CR:** Climate regulation.

**HD:** Hydrological dynamics.

**WQ:** Water quality.

**REE:** Regulation of extreme events.

**RSFE:** Regulation of soil fertility and erosion.

**RInvSp:** Regulation of invasive species, pests and diseases.

**BI:** Biotic interactions.

**BC:** Biogeochemical cycles.

**Ht:** Habitat (terrestrial).

**Ha:** Habitat (aquatic).

**Rec:** Recreational service.

**Comparison:** Type of comparison. Possible values: restored vs. degraded wetlands, restored vs. natural wetlands.

**UnitsMeas:** Measurement units.

**N (avg):** mean sample size of the different conditions (restored, degraded, and natural).

**RR:** response ratio calculated as the ln ratio of each measure of biodiversity or ecosystem service supply in restored wetlands relative to either degraded wetlands [ln(Rest/Deg] or natural wetlands [ln(Rest/Nat)]. See also the main text and **Appendix S4**.

**Var (RR):** variance in the response ratios calculated in MetaWin (see "Database criteria").

**N/A:** Information not available (i.e. missing data, see "Database criteria").

**NA:** Not applicable (see "Database criteria").

Database criteria

Each publication (**R ID**) was assigned a unique code derived from author names and publication year, as well as the letters “a” or “b” if necessary. These codes allow identification of the relevant publication in the Literature Cited section below. Since all publications reported data for more than one response variable, we included another column with the identifier **D ID** in order to identify each measurement within a study individually.

Location site (**L ID**) refers to the location or environmental conditions of a particular study.

Experimental design (**ExpDes**) was classified for each study using the following scheme:

1. BA: “before-after” design. In this design, different conditions corresponded to different samplings of the same wetland. This design included a “within-wetland” control. BA studies in our meta-analysis reported only comparisons of restored vs. degraded wetlands.
2. CI: “control-impact” design. Several wetlands in different states (restored, degraded, or natural) were compared. This design featured no “within-wetland” control. CI studies in our meta-analysis reported both a restored vs. degraded comparison and a restored vs. natural comparison.
3. PA: “paired” design. Different conditions were represented by paired-plots within the same wetland. This design was similar to the CI type except that the paired plots served as a “within-wetland” control. PA studies in our meta-analysis reported both types of comparisons.

We classified the studies into 10 widely recognized ecosystem types (**EcoType**): delta, lake, mangrove, marsh (freshwater or salt), riparian, river, stream, wet meadow, and wetland. The “wetland” category was assigned to any study that did not explicitly indicate the aquatic ecosystem type. Wetland types (**WetlType**) were defined based on Ramsar (2006). Human-made wetlands were not classified separately from naturally occurring wetlands.

We assigned the causes of degradation (**CauDeg**) operating in each wetland according to six categories (Brinson & Malvárez 2002): (a) geomorphic / hydrologic (water diversions and dams, disconnection of floodplains from flood flows, filling, and draining, water extractions); (b) nutrients / contaminants (eutrophication, loading with toxic materials, acidification); (c) invasion of exotic species (flora and fauna), (d) climate change (global warming, increased storm intensity and frequency), (e) land cover change (logging, deforestation, land conversion for agriculture or urban ecosystems), and (f) human use. The category "human use" was assigned when human activity in an ecosystem was reported or could be inferred, or when the reported causes of degradation were associated with human presence in the wetland.

We also extracted data on the specific restoration action applied in the wetland (**ResAct**). In an effort to capture the enormous diversity of these actions, we grouped them into 10 categories (**RA Group)**: (a) active revegetation (i.e. introduction of plants), (b) enhancement of in-wetland heterogeneity (i.e. any activity affecting heterogeneity of the aquatic ecosystem, such as the addition of large wood, manipulation or addition of particle size), (c) enhancement of structural heterogeneity (i.e. land contouring, terracing, meandering), (d) removal of exotic species (which turned out to be non-native vascular plants in all studies of our meta-analysis), (e) habitat creation (in our case, wetland creation, which sometimes included revegetation), (f) passive restoration (i.e. removal of degradation factors), (g) restoration of hydrological dynamics (e.g. watering, plugging drainages, and impounding water, together with revegetation in some cases), (h) restoration of water quality (i.e. water replacement, liming, nutrient or litter addition), (i) soil amendments and revegetation, and (j) wildlife management (i. e. reintroduction of native fauna and/or elimination of exotic fauna).

Restoration effects may depend on the time over which restoration actions are implemented, as well as on how many times the actions are implemented. We could not extract such data, however, because most studies implemented one-off restoration actions. Instead we collected data on the interval between when the most recent restoration action was completed and when response variables were measured (**AgeRes**). This is the only variable in our database with numerical values. Although extracted data were originally expressed in years (**Table S1**), we subsequently transformed them to ln [months] in order to include them in the linear mixed model (see main text). We did not predefine categories for the min, max, or range for this variable because it depended on the time frame of the studies.

Categories for the variables **EcoType**, **WetlType**, **RA Group**, and **AgeRes** were defined *a posteriori* according to the information extracted from the included studies. Only the classification of **CauDeg** data was defined *a priori*.

The 70 publications included in our meta-analysis reported 406 response variables (**VarMeas)**. When the same study reported multiple measurements collected over time, we collected only the latest measurements in order to avoid artifacts due to transient dynamics.

We built separate databases for meta-analyses of biodiversity and ES. In the biodiversity database, response variables (**VarMeas)** included: species, family and generic richness; diversity; evenness, including appropriate indices; and community composition, e.g. similarity indices. In studies that measured biomass, density and abundance of organisms, we defined biodiversity exclusively as changes in community structure. In both the biodiversity and ES databases, we grouped the broad diversity of taxonomic groups into the following categories: vascular plants, protista, aquatic invertebrates, macroinvertebrates, terrestrial invertebrates, nekton, amphibians, fishes, mammals. Since some studies did not clearly distinguish fish and macroinvertebrates, we were forced to aggregate them into the general category of nekton. We categorized native and exotic vascular plants separately whenever possible.

In the ES database, response variables **VarMeas** were categorized into 15 ES of wetlands (see Table 1 in the main text) (Costanza *et al*. 1997; de Groot *et al*. 2002; MEA 2005; Ramsar 2010): plant food / raw material (PF/RM); animal food / raw material (AF/RM); water supply (WS); climate regulation (CR); hydrological dynamic (HD); water quality (WQ); regulation of extreme events (REE); regulation of soil fertility and erosion (RSFE); regulation of invasive species, pests and diseases (RInvSp); biogeochemical cycling (BC); biotic interactions (BI); provision of terrestrial habitat (Ht); provision of aquatic habitat (Ha); cultural services (Cult); and recreational services (Rec). Studies reported data on stocks, flows and rates of service production. Each ES was represented by a set of binary variables such that the relationship of each measurement to each ES was clear (see main text and **Table S1**). The database contained 2,792 entries from 70 studies, reflecting the fact that many studies reported data for more than one response variable, and each combination of response variable and ES was recorded in a separate row.

These 15 ES were classified into the four major ES categories (**ES Type**) proposed by the Millennium Ecosystem Assessment (MEA 2005): supporting, regulating, provisioning, and cultural (Rey Benayas *et al*. 2009; Schmidt *et al*. 2009). Supporting services are necessary for the production of other ecosystem services. They include soil formation, photosynthesis, primary production, nutrient cycling and water cycling. Regulating services are the benefits obtained from the regulation of ecosystem processes, including air and water quality maintenance, climate regulation, erosion control, regulation of human diseases, biological control, pollination, and storm protection. Provisioning services are the products used by people that are obtained from ecosystems, including plant and animal food, fiber, fuel, genetic resources, biochemicals, natural medicines, and pharmaceuticals, decorative resources, and fresh water. Cultural services relate to human values and behavior, as well as to human institutions and patterns of social, economic, religious, and political organization.

We calculated effect sizes in terms of response ratios (RRs), which by definition are positive if the restoration increases response variables. While increases in most response variables indicate improvement, increases in others indicate degradation; for example, increases in the concentration of a contaminant in water or soil or in the abundance of non-native species imply reductions in ES provisioning. In these cases, negative RR indicates improvement in the response variable. To facilitate interpretation of RRs, we inverted the signs of negative RRs such that all improvement values would be positive. In Table S1, positive and negative signs indicate whether the response variable (**VarMeas)** is directly or inversely related, respectively, to biodiversity or ES.

Data on response variables were extracted from the text, figures and tables of the included studies. If data reporting was incomplete, we contacted the corresponding authors in an effort to obtain missing results. Data which we could not obtain from the studies or authors are marked as **N/A** (not available). The abbreviation **NA** (not applicable) identifies variables not applicable to the study in question.

For consistency, one of us (PM) extracted data, and two others (JMRB and PB) checked it. All authors jointly determined the classification and arithmetic sign of ES and biodiversity response variables.

**References**

Brinson MM, Malvárez AI (2002) Temperate freshwater wetlands: types, status, and threats. Environ Cons 29: 115-133.

Costanza RR, d'Arge R, de Groot S, Farber S, Grasso M, Hannon B, Limburg K, Naeem S, O'Neill RV, Paruelo J, Raskin RG, Sutton P, van den Belt M (1997) The value of the world's ecosystem services and natural capital. Nature 387: 253-260.

de Groot RS, Wilson MA, Boumans RMJ (2002) A typology for the classification, description and valuation of ecosystem functions, goods and services. Ecol Econ 41: 393-408.

Millennium Ecosystem Assessment (MEA) (2005) Ecosystems and Human Well-Being. Synthesis. Washington DC: Island Press. 68 p.

Ramsar (2006) The Ramsar Convention Manual: a guide to the Convention on Wetlands (Ramsar, Iran, 1971). 4th ed. Gland: Ramsar Convention Secretariat. 114 p.

Ramsar (2010) Wetland Ecosystems Services. Available: [http://www.ramsar.org/cda/en/ramsar-pubs-info-ecosystem-services/main/ramsar/1-30-103%5E24258_4000_0](http://www.ramsar.org/cda/en/ramsar-pubs-info-ecosystem-services/main/ramsar/1-30-103^24258_4000_0) Accessed 20 January of 2013.

Rey Benayas JM, Newton AC, Díaz A, Bullock JM (2009) Enhancement of biodiversity and ecosystem services by ecological restoration: a meta-analysis. Science 325: 1121-1124.

Schmid B, Pfisterer AB, Balvanera P (2009) Effects of biodiversity on ecosystem, community, and population variables reported 1974–2004. Ecol 90: 853.
