# Supplementary material for: Restoration Enhances Wetland Biodiversity and Ecosystem Service Supply, but Results Are Context-Dependent: A Meta-Analysis
Source: PLoS One. 2014 Apr 17;9(4):e93507. doi: 10.1371/journal.pone.0093507 (PMC3990551; doi:10.1371/journal.pone.0093507)
Supplement: Supporting information S3 — Methodological details of the meta-analysis. (DOC) [file pone.0093507.s004.doc]

**SUPPORTING INFORMATION S3.** Methodological details of meta-analysis.

We used the response ratio (RR) as the effect size metric (Gurevitch & Hedges 2001), which is one of the metrics most commonly used in ecology meta-analyses (Lajeunesse & Forbes 2003; Rey-Benayas *et al.* 2009). The RR was calculated for each biodiversity and ES measure as the natural logarithm-transformed ratio of the experimental value in the restored wetland to the corresponding control value in the degraded wetland [ln(Rest/Deg)] or natural wetland [ln(Rest/Nat). Such an RR relating an experimental measurement to the corresponding control measurement is often used to assess experimental effects (Hedges *et al*. 1999).

Our meta-analysis was weighted, meaning that variance and sample size were taken into account when calculating effect sizes (Gurevitch & Hedges 2001). We computed RR as X_r_/X_d_, where X_r_ is the mean value in the restored wetland and X_d_ the mean value in the control group (degraded or natural wetland). We defined Var_r_ and N_r_ as the variance and sample size (number of replicates) of the restored condition, and Var_d_ and N_d_ as the corresponding variables in the control conditions. Statistical analyses of RRs were carried out after transforming them by the natural logarithm [RR = ln(X_r_/X_d_)]. This transformation linearizes the variable, such that deviations in the numerator are treated the same as deviations in the denominator. As a result, measurements of the variable typically show a normal distribution, making the natural log-transformed RR suitable for a wide range of parametric statistical tests (Hedges *et al.* 1999).

To compute global effect size, defined as mean RR+SD for each organism or ES type, Metawin requires a single value for sample size. Since N_r_ and N_d_ were different in several studies, we averaged them together in a single variable N(avg) (**Appendix S2**). In weighted meta-analysis, the variance of the global effect size has two components: one due to sampling variation within each study and another due to variation between studies (Hedges et al. 1999). Metawin takes both components into account when computing global effect size variance, defined as Var[RR] in the database (**Appendix S2**).

To assess the statistical significance of differences in RR effect sizes among groups or the significance of deviations of effect sizes from zero, we used the conservative test of the bias-corrected 95% bootstrapping confidence intervals (CI) based on 999 permutations (Adams *et al.* 1997; Rosemberg *et al.* 2000). This approach is similar to that used in previous meta-analyses (see Hillebrand *et al.* 2007; Mooney *et al.* 2010).

The accuracy of a meta-analysis can depend on the number of studies included and on the significance of the results in those studies. Rosenthal (1979) defined the fail-safe number as the number of studies that would have to be added to a meta-analysis in order to change at least some results from significant to non-significant. He proposed that a meta-analysis can be considered reliable if the fail-safe number is larger than 5N + 10, where N is the number of studies in the dataset. In our meta-analysis, the fail-safe number for restored vs. degraded wetlands was 21,417, larger than 5N + 10 = 1,510. The fail-safe number for restored vs. natural wetlands was 3,905, again larger than 5N + 10 = 2,540. Therefore we believe that the results of our meta-analysis are reliable (Rosenberg 2005).

To assess the risk of publication bias towards positive results and larger sample sizes in our included studies, we generated funnel plots, which indicated that such bias was unlikely to affect the results of the comparisons between restored and degraded wetlands (Fig. S4.1a) or between restored and natural wetlands (Fig. S4.1b). We also plotted standardized effect sizes against the normal quantiles (Wang & Bushman 1998) to assess how well the effect sizes fit a normal distribution. Slight deviation from a normal distribution was observed (Fig. S4.2).

Since we expected the comparisons between restored vs. degraded conditions and between restored vs. natural conditions to be intrinsically different, we analyzed them separately. Since this difference may be due at least in part to differences in experimental designs, we assessed whether experimental design (ExpDes) interacted with “comparison type” (Comp) using ANOVA. These two variables were included as fixed factors and RR as the dependent variable. We confirmed the expected significant differences between comparison types (*F*_1, 661_ = 12.3, *P* = 0.0005) and between experimental designs (*F*_1, 661_ = 6.96, *P* = 0.0011), suggesting that performing separate meta-analyses for two “comparison types” is appropriate because the data sets differ numerically and ecologically. At the same time, comparison types and experimental designs did not interact significantly (*F*_1, 664_ = 2.23, *P* = 0.1375), suggesting a lack of confounding between these factors.

**References**

Adams DC, Gurevitch J, Rosenberg MS (1997). Resampling tests for meta-analysis of ecological data. Ecol 78: 1277–1283.

Gurevitch J, Hedges LV (2001) Meta – analysis: combining the results of independent experiments. In: Scheiner SM, Gurevitch J, editors. Design and analysis of ecological experiments. Oxford: Oxford University Press. pp. 347-369

Hedges LV, Gurevitch J, Curtis PD (1999) The meta-analysis of response ratios in experimental ecology. Ecol 80: 1150–1156.

Hillebrand H, Gruner DS, Borer ET, Bracken MES, Cleland EE, Elser JJ, Harpole WS, Ngai JT, Seabloom, EW, Shurin JB, Smith JE (2007) Consumer versus resource control of producer diversity depends on ecosystem type and producer community structure. Proc Nat Acad Sci 104: 10904-10909.

Lajeunesse MJ, Forbes MR (2003) Variable reporting and quantitative reviews: a comparison of three meta-analytical techniques. Ecol Lett 6: 448–454.

Mooney KA, Gruner DS, Barber NA, Van Bael SA, Philpott SM, Greenberg R (2010) Interactions among predators and the cascading effects of vertebrate insectivores on arthropod communities and plants. Proc Nat Acad Sci 107: 7335-7340.

Rey Benayas JM, Newton AC, Diaz A, Bullock JM (2009) Enhancement of biodiversity and ecosystem services by ecological restoration: a meta-analysis. Sci 325: 1121–1124.

Rosenberg MS (2005) The file-drawer problem revisited: a general weighted method for calculating fail-safe numbers in meta – analysis. Evol 59: 464-468.

Rosenberg MS, Adams DC, Gurevitch J (2000) Metawin: statistical software for meta-analysis. Sunderland: Sinauer Associates.

Rosenthal R (1979) The "file drawer problem" and tolerance for null results. Psychol Bull 86: 638-461.

Wang MC, Bushman BJ (1998) Using the normal quantile plot to explore meta-analytic data sets. Psychol Meth 3: 46-54.

Fig. S3.1. Funnel plot of effect sizes [ln(RR)] of raw data against averaged sample size in comparisons of (a) restored vs. degraded wetlands and (b) restored vs. natural wetlands.


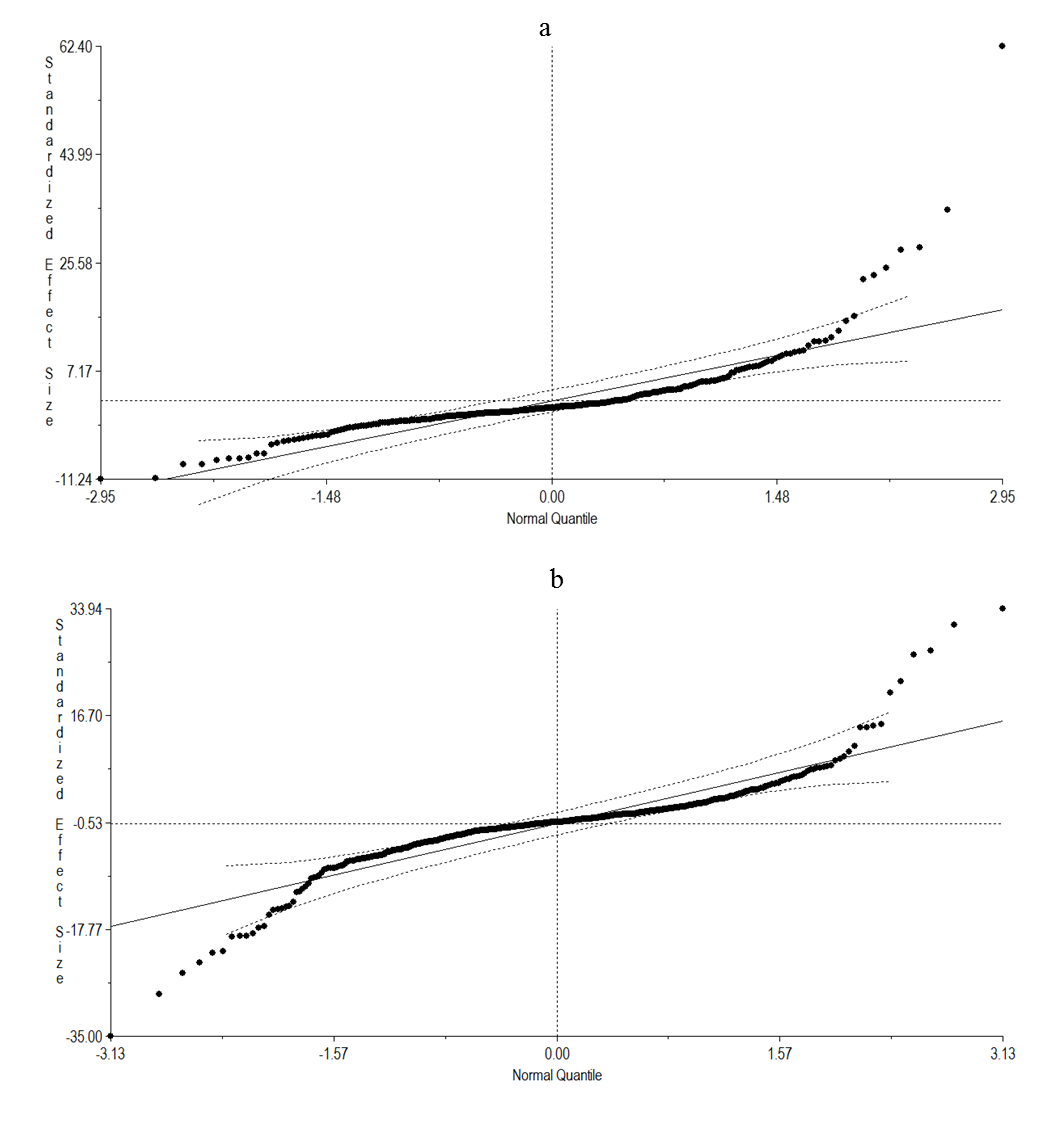


Fig. S3.2. Normal quantile plot (RR) of raw data versus sample size for comparisons of (a) restored vs. degraded wetlands and (b) restored vs. natural wetlands.
