# Supplementary material for: Restoration Enhances Wetland Biodiversity and Ecosystem Service Supply, but Results Are Context-Dependent: A Meta-Analysis
Source: PLoS One. 2014 Apr 17;9(4):e93507. doi: 10.1371/journal.pone.0093507 (PMC3990551; doi:10.1371/journal.pone.0093507)
Supplement: Supporting information S4 — Overview of studies included in the meta-analysis. (DOC) [file pone.0093507.s005.doc]

**SUPPORTING INFORMATION S4.** Overview of selected studies.

The 70 studies included in our meta-analysis analyzed 62 locations in 14 countries. A total of 49 studies were conducted in North America, including 47 in continental USA, one in Canada, and one in Alaska. Seven studies were from Europe (Spain, Sweden, Austria), five from Oceania (Australia, New Zealand), four from Asia (China), two from Africa (Kenya), one from the Western Pacific (Philippines), and one from Central America (Honduras).

The primary causes of conversion or degradation of natural ecosystems were land transformation for agricultural or urban purposes (25%) and water use practices (23%), particularly hydro-engineering including channelization, drainage, pumping and dam construction. Other, less frequent causes were over-exploitation of native species, invasion by exotic species (17%), and water quality deterioration (e.g. by eutrophication or acidification) (11%). One-quarter of the study sites were degraded by multiple causes or were assumed to be because the study did not mention specific causes of degradation.

Restoration actions included management strategies to mitigate negative effects caused by hydro-engineering, enhancement of wetland structure, habitat reclamation to benefit native species, rehabilitation of lost riverine - riparian and other vegetation types, introduction of target native species, and removal of exotic species. In 17.1% of the studies, restoration actions led to the creation of a complete wetland. Only 5% of studies involved passive restoration, even though the simplest approach for ecological restoration is to cease the degrading activity. In 10% of the studies, a combination of restoration actions was employed.

After assigning restoration actions to different classes, we determined that nearly 40% of studies dealt with hydrologic and structural characteristics of wetlands (Fig. S4.1). Several of these studies also included revegetation activities. Habitat creation was used in 19% of the studies, most of which were conducted in the USA, where wetland creation is a frequent mitigation activity. Active restoration of water or soil was reported in fewer than 10% of studies. Wildlife management actions were implemented in only 3% of studies.

Only 22.9% of the studies compared the three conditions of restored, degraded, and natural.

A total of 40% of studies reporting comparisons of restored vs. degraded wetlands used a “before-after” experimental design, compared to 90% of studies reporting comparisons of restored vs. natural wetlands. The corresponding proportions of studies using a paired design were 17% and 10%, respectively. The “control-impact” design was used in 21% of studies reporting comparisons of restored vs. degraded wetlands, and in 64% of the studies reporting comparisons of restored vs. natural wetlands.

The biodiversity database included 41 observations from 16 studies reporting comparisons of the restored vs. degraded conditions, as well as 102 observations from 25 studies reporting comparisons of restored vs. natural conditions.

The ES database contained 259 observations from 27 studies reporting comparisons of restored vs. degraded conditions. Among these studies, the largest numbers of observations were related to supporting services (125) and regulating services (119), while substantially fewer observations were available for provisioning services (10) and cultural services (5). The database also included 407 observations from 37 studies reporting comparisons of restored vs. natural wetlands. Again, the largest numbers of observations were related to supporting services (188) and regulating services (190), with many fewer observations related to provisioning services (22) and cultural services (7).

Fig. S4.1. Distribution of restoration actions among the 62 sites in the 70 studies included in our meta-analysis. Act Rev, active revegetation; In-wetland heterog, enhancement of in-wetland heterogeneity; Struc heterog, enhancement of structural heterogeneity; Exotic sp removal, removal of exotic species; Hab creation, habitat creation; Pass Res, passive restoration; Hydrol Dyn, restoration of hydrological dynamics; Water qual, restoration of water quality; Soil & Reveg, soil amendment and active revegetation; Wildlife, wildlife management. See Appendix S2 in Supporting Material for details about restoration actions.
