# Supplementary material for: Restoration Enhances Wetland Biodiversity and Ecosystem Service Supply, but Results Are Context-Dependent: A Meta-Analysis
Source: PLoS One. 2014 Apr 17;9(4):e93507. doi: 10.1371/journal.pone.0093507 (PMC3990551; doi:10.1371/journal.pone.0093507)
Supplement: Figure S5 — PRISMA 2009 Flow Diagram. (DOC) [file pone.0093507.s010.doc]

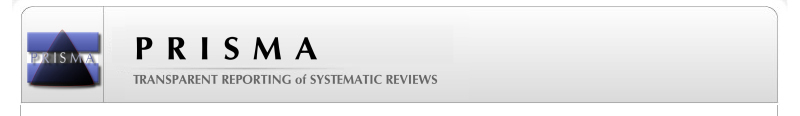
**PRISMA 2009 Flow Diagram**

**Screening**

**Included**

**Eligibility**

**Identification**

Records identified through database searching
(n = 2769)

Additional records identified through other sources
(n = 0)

Records after duplicates removed
(n = 2717)

Records screened
(n = 1937)

Records excluded
(n = 1852)

Full-text articles assessed for eligibility
(n = 85)

Full-text articles excluded, with reasons
(n = 15)

Studies included in qualitative synthesis
(n = 70)

Studies included in quantitative synthesis (meta-analysis)
(n = 70)
