# Supplementary material for: Restoration Enhances Wetland Biodiversity and Ecosystem Service Supply, but Results Are Context-Dependent: A Meta-Analysis
Source: PLoS One. 2014 Apr 17;9(4):e93507. doi: 10.1371/journal.pone.0093507 (PMC3990551; doi:10.1371/journal.pone.0093507)
Supplement: Table S2 — Comparison of biodiversity meta-analyses using a reduced or complete database. (DOC) [file pone.0093507.s012.doc]

**Table S2** Sample sizes (N), effect sizes (RR) and bias-corrected 95% bootstrapping confidence intervals (Bias CI) of RRs calculated for three types of organism (namely macroinvertebrates, aquatic invertebrates, and vascular plants) when taking into account all effect sizes (whole dataset) or only one effect size per study (reduced dataset).

|  |  | *Restored vs. Degraded Wetlands* | | | *Restored vs. Natural Wetlands* | | |
| --- | --- | --- | --- | --- | --- | --- | --- |
|  | Dataset | N | RR | Bias CI | N | RR | Bias CI |
| Biodiversity |  |  |  |  |  |  |  |
| Vascular Plants |  |  |  |  |  |  |  |
|  | Reduced | 5 | 0.6102 | 0.2136 to 0.9161 | 10 | 0.1627 | -0.0203 to 0.4210 |
|  | Whole | 9 | 0.4382 | 0.1279 to.8615 | 43 | -0.025 | -0.1895 to 0.1477 |
| Macroinvertebrates |  |  |  |  |  |  |  |
|  | Reduced | 4 | -0.0121 | -0.1251 to 0.1159 | 5 | -0.1143 | -0.1820 to -0.0190 |
|  | Whole | 9 | -0.0029 | -0.0526 to 0.0507 | 16 | -0.0497 | -0.1250 to 0.0302 |
| Aquatic invertebrates |  |  |  |  |  |  |  |
|  | Reduced | 8 | 0.2233 | -0.1306 to 0.8531 | 7 | -0.057 | -0.3637 to 0.2544 |
|  | Whole | 19 | 0.2173 | 0.0202 to 0.3851 | 15 | -0.0815 | -0.2301 to 0.0880 |
